# Complete Mitochondrial Genome of *Tanypus chinensis* and *Tanypus kraatzi* (Diptera: Chironomidae): Characterization and Phylogenetic Implications

**DOI:** 10.3390/genes15101281

**Published:** 2024-09-29

**Authors:** Shaobo Gao, Chengyan Wang, Yaning Tang, Yuzhen Zhang, Xinyu Ge, Jiwei Zhang, Wenbin Liu

**Affiliations:** 1 Yinshanbeilu Grassland Eco-Hydrology National Observation and Research Station, China Institute of Water Resources and Hydropower Research, Beijing 100038, China; gaoshaobo@caas.cn; 2 Grassland Research Institute of Chinese Academy of Agricultural Science, Hohhot 010010, China; 3 Tianjin Key Laboratory of Conservation and Utilization of Animal Diversity, Tianjin Normal University, Tianjin 300387, Chinaskygxy@tjnu.edu.cn (X.G.); 4 College of Prataculture, Qingdao Agricultural University, Qingdao 266109, China; 5 Changjiang Basin Ecology and Environment Monitoring and Scientific Research Center, Changjiang Basin Ecology and Environment Administration, Ministry of Ecology and Environment, Wuhan 430010, China

**Keywords:** Chironomidae, mitogenome, phylogenomics, *Tanypus*

## Abstract

Background: Chironomidae occupy a pivotal position within global aquatic ecosystems. The unique structural attributes of the mitochondrial genome provide profound insights and compelling evidence, underpinning the morphological classification of organisms and substantially advancing our understanding of the phylogenetic relationships within Chironomidae. Results: We have meticulously sequenced, assembled, and annotated the mitogenomes of *Tanypus chinensis* (Wang, 1994) and *Tanypus kraatzi* (Kieffer, 1912), incorporating an additional 25 previously published mitogenomes into our comprehensive analysis. This extensive dataset enables us to delve deeper into the intricate characteristics and nuances of these mitogenomes, facilitating a more nuanced understanding of their genetic makeup. Conclusions: The genomic nucleotide composition of *T. kraatzi* was 39.10% A, 36.51% T, 14.33% C, and 10.06% G, with a total length of 1508 bp. The genomic nucleotide composition of *T. chinensis* was 39.61% A, 36.27% T, 14.55% C, and 9.57% G, with a total length of 1503 bp. This significant enrichment of the chironomid mitogenome library establishes a novel foundation for further exploration in the realm of phylogenetics.

## 1. Introduction

Chironomidae, a group of Diptera insects belonging to the suborder of Nematocera, known as non-biting midges in adults and bloodworms during the larval stage, occupy a pivotal position within global aquatic ecosystems [1,2]. Their unparalleled species diversity and remarkable resilience to diverse environmental fluctuations render them an exemplary model for delving into the mechanisms of genetic adaptability among aquatic insects [3]. The Chironomidae family, comprising an extensive array of 11 sub-families, boasts an impressive tally of over 6200 species [2]. Among these, the subfamilies of Orthocladiinae, Tanypodinae, and Chironominae stand out, housing the largest concentration of species and exhibiting a widespread distribution across the globe [4].

*Tanypus*, erected by Meigen in 1803, serves as the type genus of the Tanypodinae subfamily [5]. The larvae of those species reside in sediments found within stagnant and slowly flowing water bodies, particularly in temperate to warm climatic zones [6]. Remarkably, they exhibit a remarkable tolerance to high nutrient concentrations and varying degrees of salinity in these environments [6]. Currently, there are 30 species recorded globally within this genus, with only 3 species documented in China [5,7]. *Tanypus punctipennis* Meigen 1818 is widely distributed worldwide, including many regions in China [5]. *Tanypus formosaus* (Kieffer, 1912) is restricted to the distribution in Taiwan Province, China [8]. *Tanypus chinensis* Wang 1994 is currently restricted to a limited distribution in China within regions such as Guizhou Province, the Inner Mongolia Autonomous Region, Hunan Province, Liaoning Province, and Hebei Province [8]. *Tanypus kraatzi* (Kieffer, 1912) is a widely distributed species in the Palearctic region, yet there are currently no recorded occurrences of it within China. The first record of this species in China can be found in [5].

Recently, insect mitochondrial genomes have garnered substantial research attention, showcasing an extraordinary degree of conservation in their intricate structural architecture [9]. This remarkable feature underscores their significance as a subject of intense scientific scrutiny, revealing insights into the evolutionary history and functional mechanisms of these vital cellular organelles [9,10]. The distinctive attributes of the mitochondrial genome provide invaluable insights and robust evidence, profoundly enriching our comprehension of morphological classification [11]. This, in turn, contributes immensely to the advancement of Chironomidae phylogeny studies, offering a deeper understanding of the evolutionary relationships and diversity within this fascinating insect group [12,13]. However, there are relatively few reports on the mitochondrial genomes of the subfamily Tanypodinae, with only two species, *Clinotanypus yani* (Cheng and Wang, 2008) and *T. punctipennis* Meigen 1818 having their mitochondrial genomes annotated [13,14].

To gain more information on the mitochondrial genomes of the subfamily Tanypodinae, and to gain insights into the internal phylogenetic relationships within the Chironomidae, here we have sequenced, assembled, and annotated the mitogenomes of *T. chinensis* (Wang, 1994) and *T. kraatzi* (Kieffer, 1912). Adult males of those two species are readily characterized by the possession of an ovoid scutal tubercle. Furthermore, to gain a deeper understanding of the mitogenome characteristics, we integrated 25 previously published mitogenomes into our analysis. Employing robust Bayesian Inference (BI) and Maximum Likelihood (ML) methods across diverse databases, we reconstructed the intricate phylogenetic relationships among the subfamilies Tanypodinae, Podonominae, Diamesinae, Prodiamesinae, Chironomidae, and Orthocladiinae. This comprehensive analysis, encompassing 27 mitochondrial genomes, was augmented by selecting the family Ceratopogonidae as outgroups, providing valuable insights into the evolutionary history of these taxa.

## 2. Materials and Methods

### 2.1. Sampling and Sequencing

Samples of *T. chinensis* (three adult males) were collected from Huanghuagou Scenic Area, Wulanchabu City, Inner Mongolia Autonomous Region of China (112°52′91″ E, 41°13′30″ N) on 23 July 2018, and *T. kraatzi* (two adult males and one larva) from Lakes in Taizhou University, Taizhou City, Zhejiang Province of China (121°38′98″ E, 28°65′29″ N) on 14 July 2010. Species identification relies heavily on a dual approach encompassing morphological assessment and barcode comparison, with the morphological traits of the two species in question adhering to the descriptions outlined in [15,16]. The genomic DNA was meticulously extracted from the thorax and leg tissues, utilizing the Qiagen DNA Blood and Tissue Kit at Tianjin Normal University (TJNU), Tianjin, China, adhering to the standardized protocol. Prior to the DNA extraction process and morphological examination, all samples underwent a preservation step in a solution of 85% ethanol since the time of collection, maintained at a temperature of −20 °C. These voucher specimens have been archived at the College of Life Sciences, TJNU, in Tianjin, China, ensuring their availability for future reference and analytical endeavors.

To amplify the standard 658 bp mitochondrial cytochrome c oxidase subunit I (COI) barcode region, we employed universal primers LCO1490 and HCO2198, adhering to the methodologies established in [17,18]. Subsequently, the entire genome sequences were entrusted to Berry Genomics, Beijing, China, for high-throughput sequencing. The Illumina Truseq Nano DNA HT Sample Preparation Kit (USA) facilitated the preparation of sequencing libraries, ensuring optimal conditions for downstream analyses.

Utilizing the Illumina Nova 6000 platform (PE150), we sequenced DNA fragments with an insert size of 350 bp, adopting a paired-end strategy that maximized data throughput. The raw sequencing reads were then subjected to rigorous quality control, where Trimmomatic was employed to trim and clean the data, eliminating low-quality sequences and artifacts. The resulting high-quality, clean reads were subsequently utilized for downstream bioinformatics analyses [19], marking the first step towards elucidating the genetic makeup and evolutionary relationships of these species.

### 2.2. Assembly, Annotation, and Composition Analyses

To reconstruct the mitogenome sequences from scratch, we leveraged NOVOPlasty v3.8.3 (Brussels, Belgium), utilizing the COI barcoding sequence as the initial seed and experimenting with a diverse range of k-mer sizes, spanning from 23 to 39 bp, to optimize the assembly process [20]. The annotation of the assembled mitogenome adhered to the rigorous methodology outlined in [13], ensuring accurate identification of functional elements. The secondary structure of tRNAs was meticulously examined using the MITOS 2 WebServer, providing insights into their conformational features. For the annotation of rRNAs and Protein-Coding Genes (PCGs), we adopted a hybrid approach, initially leveraging the Clustal Omega algorithm within Geneious for automated annotations, followed by manual refinement to ensure accuracy. Furthermore, the Clustal W function integrated within MEGA 11 was employed as an additional layer of verification, refining the boundaries of rRNAs and PCGs [21,22]. To gain insights into the nucleotide composition and biases within the mitogenome, we utilized SeqKit v0.16.0, a powerful tool developed in Chongqing, China [23]. This analysis revealed not only the overall nucleotide composition but also the specific composition of each gene. The visual representation of the mitogenome was crafted using the CGView server URL: https://cgview.ca/ (accessed on 25 March 2024), providing a comprehensive and intuitive overview of the genetic architecture. To delve deeper into the codon usage patterns, we employed MEGA 11 [24], which facilitated the calculation of nucleotide composition, codon usage, and relative synonymous codon usage. Additionally, we quantified the bias in nucleotide composition using the AT-skew, defined as (A − T)/(A + T), and the GC-skew, calculated as (G − C)/(G + C), offering insights into the potential evolutionary pressures shaping the mitogenome. Lastly, to understand the evolutionary dynamics of the mitogenome, we computed the synonymous (Ks) and non-synonymous substitution rates (Ka) using DnaSP6 [25]. This analysis shed light on the selective pressures acting on the mitogenome, distinguishing between changes that alter amino acid sequences (non-synonymous) and those that do not (synonymous).

**Table 1:** Mitogenomes of the 27 species used in this study.

Family	Species	GenBank Accession Number	Reference
Chironomidae	*Clinotanypus yani*	MW373524	[2]
	*Tanypus chinensis*	PQ014462	This study
	*Tanypus punctipennis*	MZ475054	[14]
	*Tanypus kraatzi*	PQ014453	This study
	*Parochlus steinenii*	NC027591	NCBI
	*Diamesa tonsa*	MZ158292	[11]
	*Diamesa loefferi*	MZ127838	[11]
	*Sympotthastia takatensis*	MZ231026	[11]
	*Boreoheptagyia kurobebrevis*	MZ043576	[11]
	*Boreoheptagyia zhengi*	OM302508	[11]
	*Prodiamesa olivacea*	MW373525	[26]
	*Monodiamesa* sp.	MW837769	[26]
	*Monodiamesa bonalpicola*	MW837770	[26]
	*Propsilocerus akamusi*	MW846253	[26]
	*Propsilocerus taihuensis*	MW837766	[26]
	*Chironomus kiiensis*	ON838253	[27]
	*Chironomus plumosus*	ON838252	[27]
	*Stenochironomus okialbus*	OL753645	[12]
	*Stenochironomus gibbus*	OL742440	[12]
	*Polypedilum heberti*	OP950225	[12]
	*Polypedilum nubifer*	MZ747090	[27]
	*Cricotopus trifasciatus*	OP006250	[28]
	*Cricotopus triannulatus*	OP006254	[28]
	*Thienemanniella tusimufegea*	OR333983	[29]
	*Thienemanniella triangula*	OR333981	[29]
Ceratopogonidae	*Forcipomvia pulchrithorax*	NC084322	[30]
	*Forcipomyia makanensis*	MK000395	[31]

### 2.3. Phylogenetic Analyses

To delve into the phylogenetic positioning of *T. chinensis* and *T. kraatzi*, mitochondrial genome sequences of 27 registered Chironomidae species were retrieved from GenBank at NCBI in Table 1. This comprehensive dataset encompassed six Chironominae species, five Diamesinae species, four Orthocladiinae species, five Prodiamesinae species, two Tanypodinae species, and one Podonominae species. *Forcipomyia makanensis* and *Forcipomyia pulchrithorax* (Diptera: Ceratopogonidae: Forcipomyiinae) were used as an outgroup (Table 1). For conducting phylogenetic analysis, a meticulous selection of 27 mitochondrial genomes was made, from which 2 rRNAs and 13 Protein-Coding Genes (PCGs) were extracted. To align these sequences accurately, MAFFT (Osaka, Japan) was utilized, adopting the L-INS-I method to eliminate ambiguous alignment regions for both nucleotide and protein sequences in a batch process. Following alignment, Trimal v1.4.1 (Barcelona, Spain) was applied to refine the alignments by trimming, ensuring high-quality data for further phylogenetic analyses. Five distinct data matrices were generated using FASconCAT-G v1.04 (Santa Cruz, CA, USA), each tailored to capture different aspects of the genetic information. PCG Matrix: comprising all three codon positions of the 13 PCGs, providing a comprehensive view of the coding region. PCG_RNA Matrix: enlarging the scope to include both the 13 PCGs (all codon positions) and the 2 rRNAs, integrating both coding and non-coding elements. PCG12_RNA Matrix: selectively incorporating the first and second codon positions of the PCGs alongside the rRNAs, focusing on the most conserved regions of the coding genes. PCG12 Matrix: narrowing down to just the first and second codon positions of the 13 PCGs, emphasizing the evolutionary signal within these key positions. PCG_AA Matrix: utilizing the amino acid sequences derived from the 13 PCGs, abstracting away from nucleotide-level variations to assess protein-level relationships. To evaluate the heterogeneity among these diverse matrices, AliGROOVE v1.06 (Bonn, Germany) was engaged, leveraging insights from previous studies [17,32,33] as benchmarks. Subsequently, two phylogenetic trees were constructed: a Maximum Likelihood (ML) tree using IQ-tree v2.0.7, and a Bayesian Inference (BI) tree utilizing Phylobayes-MPI v1.8. These analyses offered robust insights into the evolutionary relationships among the mitochondrial genomes under study.

## 3. Results

**Figure 1:**
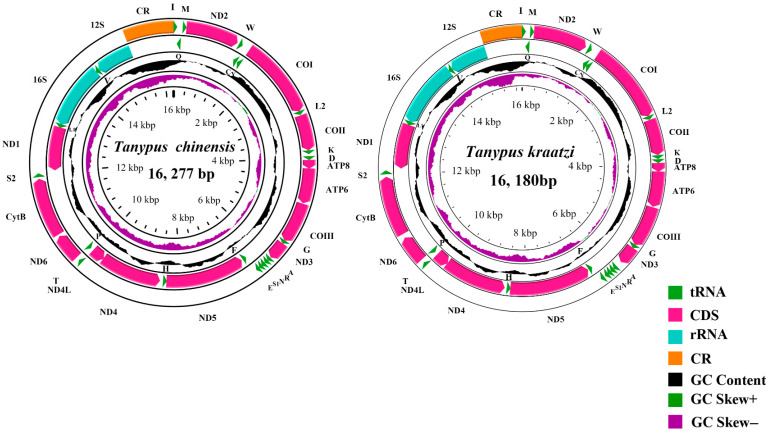
The mitogenome map depicted the distinctive mitochondrial genome attributes of various representative species spanning two genera within the *Tanypus*. The arrow directing the viewer’s gaze shows gene transcription direction. We used standardized abbreviations for PCGs and rRNAs and concise notations for tRNAs for clarity. The second circle highlighted GC content, revealing nucleotide composition. The third circle showed GC-skew, enhancing the understanding of structural asymmetry. The innermost circle summarized mitogenome length, offering a holistic view of its characteristics.

The complete mitogenome of *T. kraatzi* was 16,180 bp and *T. chinensis* was 16,266 bp long. They consist of 13 Protein-Coding Genes (PCGs), 22 tRNA genes, 2 rRNA genes (totaling 37 genes), and 1 Control Region (Figure 1).

The genomic nucleotide composition of *T. kraatzi* was 39.10% A, 36.51% T, 14.33% C, and 10.06% G, with an A + T bias of 75.61%. The total length of the 13 PCGs in the mitochondrial genome was 11,216 bp. Most PCGs start with ATN codon excluding *COX1* (*ACG*) and *ND5* (*GTG*). The length of the tRNA genes ranged from 63 to 72 bp (Figure S1), with a total length of 1508 bp. The lengths of the 12S rRNA and 16S rRNA were 811 and 1382 bp, respectively (Table 2).

The genomic nucleotide composition of *T. chinensis* was 39.61% A, 36.27% T, 14.55% C, and 9.57% G, with an A + T bias of 75.88%. The total length of the 13 PCGs in the mitochondrial genome was 11,216 bp. Most PCGs start with ATN codon excluding *COX1* (*ACG*), *ND,1* and *ND5* (*GTG*). The length of the tRNA genes ranged from 64 to 72 bp (Figure S2), with a total length of 1503 bp. The lengths of the 12S rRNA and 16S rRNA were 807 and 1395 bp (Table 3).

**Figure 2:**
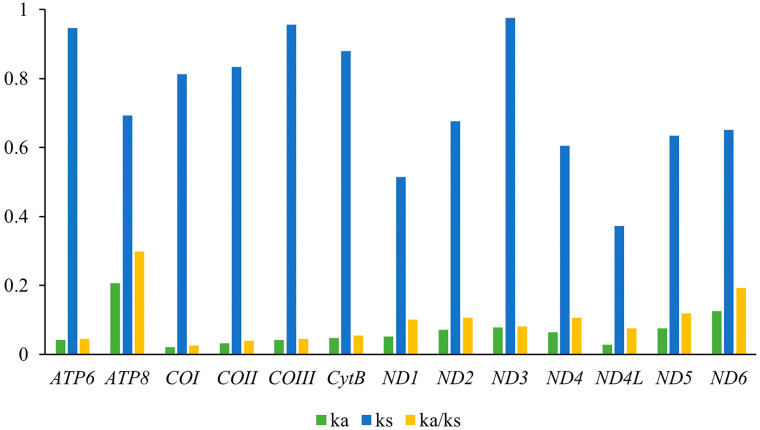
The evolution rate of 13 PCGs of the *Tanypus* mitogenomes. Ka refers to non-synonymous nucleotide substitutions, Ks refers to synonymous nucleotide substitutions, and Ka/Ks refers to the selection pressure of each PCG. The abscissa represented 13 PCGs, and the ordinate represented Ka/Ks values.

The Ka/Ks ratio (ω), a metric for quantifying evolutionary sequence rates under natural selection, consistently fell below one across all 13 Protein-Coding Genes (PCGs) in our study, mirroring trends in other insect species. Varying from 0.025 (*COX1*) to 0.299 (*ATP8*), these rates suggest varying levels of purifying selection, with ATP8 evolving fastest and *COX1* slowest (Figure 2). Genes under stronger purifying selection, like *COX2* and *COX1*, exhibit lower ω values, while *ATP8*, *ND6*, and *ND5* reflect a more relaxed selection pressure. These findings underscore the role of natural selection in shaping PCG evolution.

**Figure 3:**
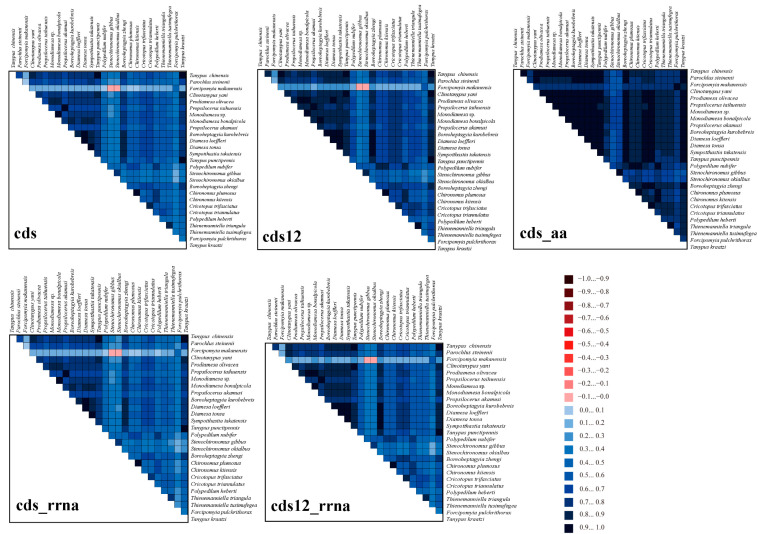
The assessment of the heterogeneity among the mitogenomes of 27 species belonging to the Chironomidae and Ceratopogonidae. Emphasizing Protein-Coding Genes (PCGs), amino acid sequences, and ribosomal RNAs (rRNAs), we visually represented sequence similarity through colored blocks. AliGROOVE scores ranging from −1 (red, for significant heterogeneity) to +1 (blue, for minimal heterogeneity) were applied. Lighter hues indicate higher heterogeneity, while darker tones signify less.

The analysis of heterogeneity divergence differences provides a window into the similarities existing in mitochondrial gene sequences across distinct species. Notably, owing to the degeneracy of codons, the dataset AA exhibited the least heterogeneity, whereas the cds12_rRNA dataset displayed a relatively higher degree of heterogeneity (Figure 3).

## 4. Discussion

**Figure 4:**
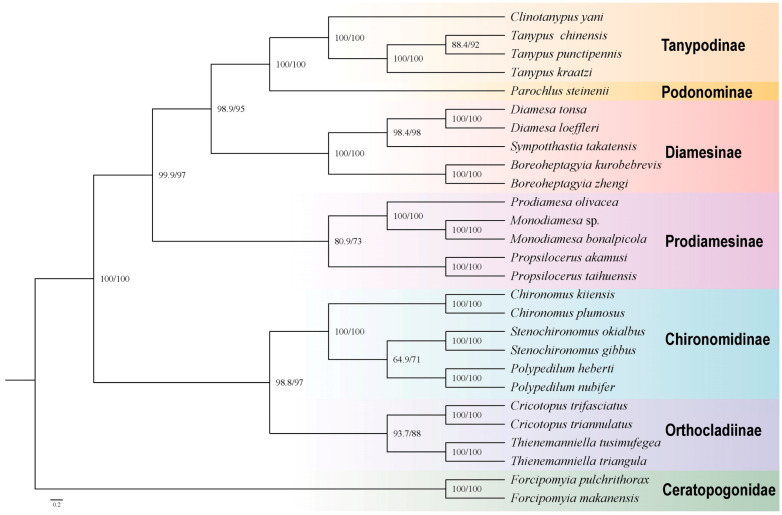
Phylogenetic tree of Chironomidae, ML tree based on analysis cds_rRNA in Partition.

This observation suggests that the mutation rate of the third codon in Protein-Coding Genes (PCG) surpassed that of the first and second codons. Consequently, the positions of the third codons were deemed unsuitable for reconstructing the phylogenetic relationship among the three genera. In our study, we harnessed the power of Bayesian Inference (BI) and Maximum Likelihood (ML) methods, utilizing five distinct matrices that culminated in the generation of ten phylogenetic trees. Our data revealed that *T. kraatzi* and *T. chinensis* belonged to the Tanypodinae and were close to the *C. yani* (Figure 4). They reveal that *T. chinensis* is a sister taxon to *T. punctipennis*, and *T. kraatzi* is a sister taxon to (*T. punctipennis* + *T. chinensis*).

In our study, the subfamilies of Tanypodinae and Podonominae were covered to be sister groups, which is consistent with previous morphological-based studies on the internal phylogenetic relationships within the Chironomidae [34,35,36,37,38]. Similarly, phylogenetic analyses based on several short sequence fragments also supported the two subfamilies as sister groups [39]. However, upon extensive sampling, the status of Tanypodinae and Podonominae as sister groups was not supported in subsequent studies such as [4].

The subfamilies Diamesinae and Prodiamesinae were found to be more closely related to the subfamilies Orthocladiinae and Chironomidiinae. Based on morphology, fragments such as COI I, CAD, 18S RNA, and 28S RNA were analyzed, and even studies based on mitochondrial genomes revealed a similar structure [4,25,39]. However, after we expanded the mitochondrial genomic information for species within the subfamilies Diamesinae and Prodiamesinae, the relationship between (((Tanypodinae + Podonominae) + Diamesinae) + Prodiamesinae) and (Orthocladiinae + Chironomidiinae) emerged as a sister group.

In our study, the subfamilies Tanypodinae and Podonominae were considered sister groups, which is largely consistent with previous research findings. Furthermore, Diamesinae was found to be a sister group to (Tanypodinae + Podonominae), resulting in the relationship between (((Tanypodinae + Podonominae) + Diamesinae) + Prodiamesinae) and (Orthocladiinae + Chironomidiinae) emerging as a sister group. This observation is inconsistent with previous results where Diamesinae and Prodiamesinae were based on a limited number of species referring to mitochondrial analysis, thereby offering new insights into the phylogenetic relationships within Chironomidae.

## 5. Conclusions

The mitochondrial genomes of the genus *Tanypus*, including those of two species, have been annotated, assembled, and reported for the first time. All newly sequenced mitogenomes had similar structural characters and nucleotide compositions to previously published Chironomidae data. This significant enrichment of the chironomid mitogenome library establishes a novel foundation for further exploration in the realm of phylogenetics.

Given the characteristic differences observed among the larvae, pupae, male, and female adults within different subfamilies of Chironomidae, there exist conflicts between phylogenetic results derived from morphology, short gene fragments, and even mitochondrial genome data. Nonetheless, a growing body of evidence from molecular biology-based phylogenetic relationships underscores the continuing significance of morphological analysis in Chironomid studies. Furthermore, although complete mitochondrial genomic analysis holds potential promise, it requires heightened scrutiny and critical attention. A comprehensive systematic analysis that integrates morphological, biogeographical, and life history characteristics across different life stages of insects, along with genomic data, is necessary and may provide insights into the natural evolutionary relationships.

## Figures and Tables

**Table 2:** Nucleotide composition and skewness of mitogenomes of *T. chinensis* (PCG: Protein-Coding Gene, CR: Control Region).

Gene Type	Length (bp)	Base Composition (%)						Skew	
		A	T	C	G	A + T	G + C	AT-Skew	GC-Skew
Whole genome	16,266	39.61	36.27	14.55	9.57	75.88	24.12	0.044	−0.206
PCG	11,216	31.28	42.73	13.45	12.54	74.00	26.00	−0.155	−0.035
PCG 1st codon position	3740	31.96	36.47	12.22	19.35	68.43	31.57	−0.066	0.226
PCG 2nd codon position	3738	20.99	45.57	19.93	13.51	66.56	33.44	−0.369	−0.192
PCG 3rd codon position	3738	40.87	46.15	8.21	4.77	87.03	12.98	−0.061	−0.265
*ATP6*	678	32.45	41.00	15.63	10.91	73.45	26.54	−0.116	−0.178
*ATP8*	168	42.86	39.88	12.50	4.76	82.74	17.26	0.036	−0.448
*COX1*	1534	28.68	37.87	17.67	15.78	66.55	33.45	−0.138	−0.057
*COX2*	688	35.03	37.79	15.12	12.06	72.82	27.18	−0.038	−0.113
*COX3*	789	30.54	36.88	17.74	14.83	67.42	32.57	−0.094	−0.089
*CYTB*	1137	32.63	37.03	17.77	12.58	69.66	30.35	−0.063	−0.171
*ND1*	948	24.58	49.05	9.07	17.30	73.63	26.37	−0.332	0.312
*ND2*	1026	32.46	45.42	12.87	9.26	77.88	22.13	−0.166	−0.163
*ND3*	354	31.07	41.81	16.38	10.73	72.88	27.11	−0.147	−0.208
*ND4*	1341	28.34	47.35	8.58	15.73	75.69	24.31	−0.251	0.294
*ND4L*	294	27.55	52.04	6.80	13.61	79.59	20.41	−0.308	0.334
*ND5*	1734	28.43	45.50	9.69	16.38	73.93	26.07	−0.231	0.257
*ND6*	525	34.48	46.48	12.19	6.86	80.96	19.05	−0.148	−0.280
All rRNA	2202	37.30	42.91	6.73	13.07	80.21	19.79	−0.070	0.320
*12S*	807	36.68	42.38	7.43	13.51	79.06	20.94	−0.072	0.290
*16S*	1395	37.92	43.44	6.02	12.62	81.36	18.64	−0.068	0.354
CR	952	47.16	43.59	7.14	2.10	90.75	9.24	0.039	−0.545
tRNA	1503	38.59	37.59	9.98	13.84	76.18	23.82	0.013	0.162

**Table 3:** Nucleotide composition and skewness of mitogenomes of *T. kraatzi* (PCG: Protein-Coding Gene, CR: Control Region).

Gene Type	Length (bp)	Base Composition (%)						Skew	
		A	T	C	G	A + T	G + C	AT-Skew	GC-Skew
Whole genome	16,180	39.10	36.51	14.33	10.06	75.61	24.39	0.034	−0.175
PCG	11,216	31.13	43.19	12.98	12.70	74.32	25.68	−0.162	−0.011
PCG 1st codon position	3740	32.55	37.08	11.56	18.82	69.62	30.38	−0.065	0.239
PCG 2nd codon position	3738	20.63	45.64	19.78	13.95	66.27	33.73	−0.377	−0.173
PCG 3rd codon position	3738	40.20	46.87	7.60	5.33	87.07	12.93	−0.077	−0.176
*ATP6*	678	29.94	40.27	16.67	13.13	70.21	29.80	−0.147	−0.119
*ATP8*	168	41.67	38.69	12.50	7.14	80.36	19.64	0.037	−0.273
*COX1*	1534	29.01	37.09	17.99	15.91	66.10	33.90	−0.122	−0.061
*COX2*	688	34.74	36.63	15.84	12.79	71.37	28.63	−0.026	−0.107
*COX3*	789	29.66	37.90	16.86	15.59	67.56	32.45	−0.122	−0.039
*CYTB*	1137	31.75	37.99	17.24	13.02	69.74	30.26	−0.089	−0.139
*ND1*	948	24.58	49.05	8.97	17.41	73.63	26.38	−0.332	0.320
*ND2*	1026	32.46	45.22	12.48	9.84	77.68	22.32	−0.164	−0.118
*ND3*	354	31.64	41.81	15.25	11.30	73.45	26.55	−0.138	−0.149
*ND4*	1341	28.11	47.20	9.40	15.29	75.31	24.69	−0.253	0.239
*ND4L*	294	25.85	52.38	8.16	13.61	78.23	21.77	−0.339	0.250
*ND5*	1734	29.01	45.67	9.57	15.74	74.68	25.31	−0.223	0.244
*ND6*	525	33.71	48.95	10.67	6.67	82.66	17.34	−0.184	−0.231
All rRNA	2193	38.06	42.08	7.17	12.70	80.13	19.87	−0.050	0.279
*12S*	811	37.98	40.81	7.89	13.32	78.79	21.21	−0.036	0.256
*16S*	1382	38.13	43.34	6.44	12.08	81.47	18.52	−0.064	0.305
CR	781	44.81	46.73	6.40	2.05	91.54	8.45	−0.021	−0.515
tRNA	1508	37.86	38.46	10.21	13.46	76.32	23.67	−0.008	0.137

## Data Availability

All data are available in the manuscript or the Supplementary Materials.

## Supplementary Materials

The following supporting information can be downloaded at https://www.mdpi.com/article/10.3390/genes15101281/s1, Figure S1: Putative secondary structures of the 22 tRNA genes identified in the mitogenome of *Tanypus chinensis*; Figure S2: Putative secondary structures of the 22 tRNA genes identified in the mitogenome of *Tanypus kraatzi*.
