# Supplementary figures and images for: Complete Mitochondrial Genome of Tanypus chinensis and Tanypus kraatzi (Diptera: Chironomidae): Characterization and Phylogenetic Implications
Source: Genes (Basel). 2024 Sep 29;15(10):1281. doi: 10.3390/genes15101281 (PMC11508139; doi:10.3390/genes15101281)

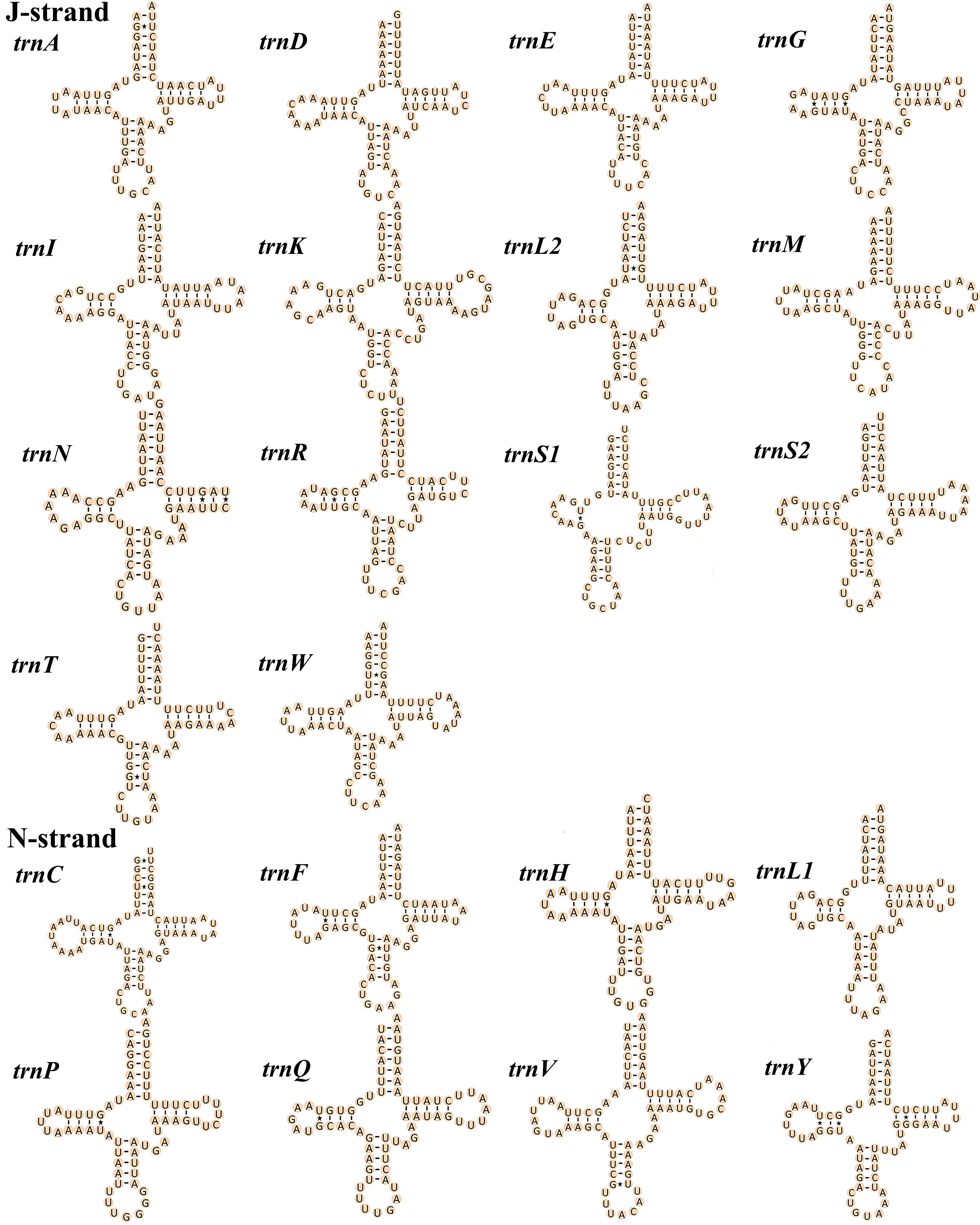

Supplement: Supplementary file 1 [file genes-15-01281-s001.zip › Tanypus kraatzi.jpg]

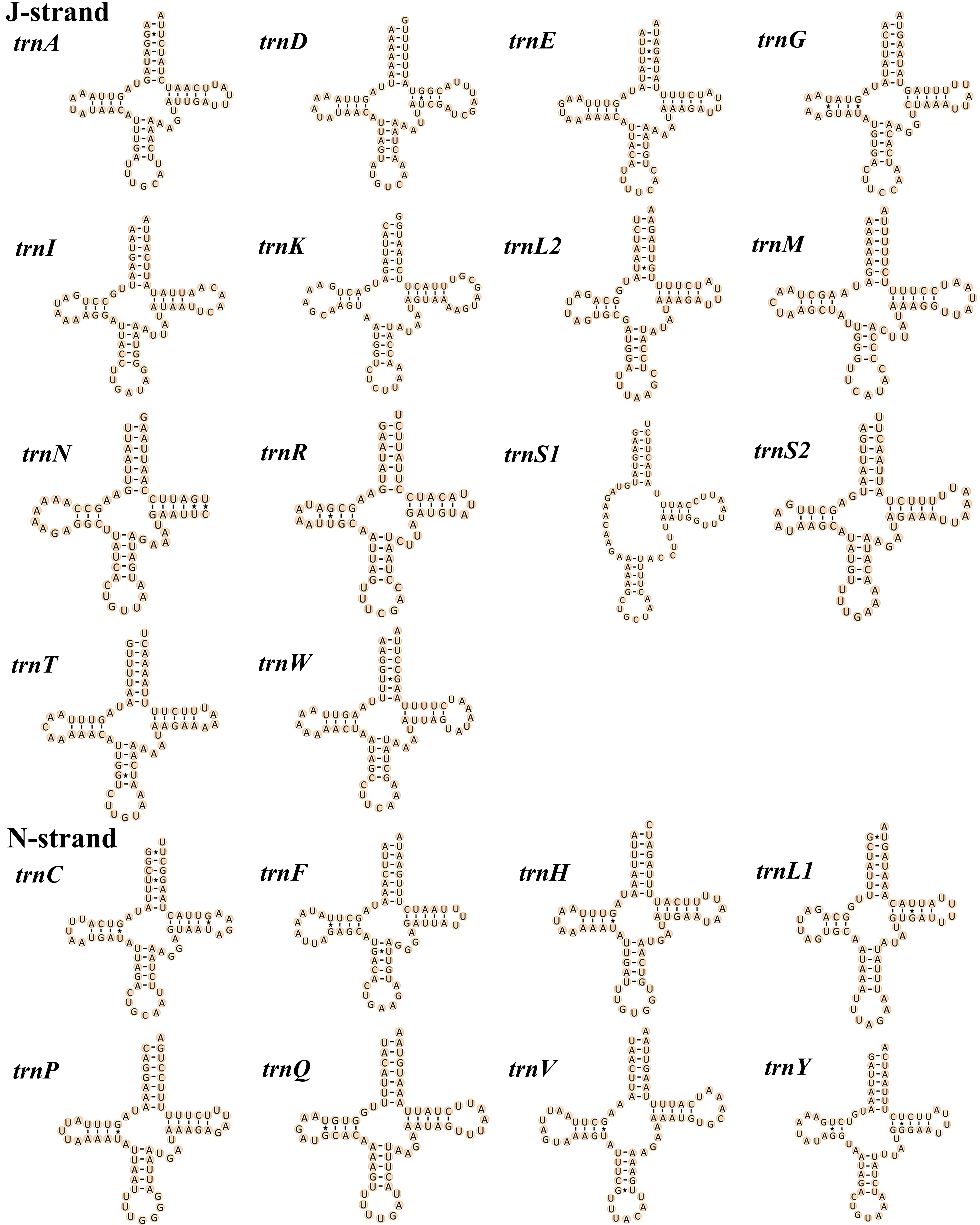

Supplement: Supplementary file 1 [file genes-15-01281-s001.zip › Tanypus chinensis.jpg]
